# Frequency of Direct Funduscopy Upon Initial Encounters for Patients with Headaches, Altered Mental Status, and Visual Changes: A Pilot Study

**DOI:** 10.3389/fneur.2015.00233

**Published:** 2015-11-09

**Authors:** Esteban Golombievski, Michael W. Doerrler, Sean D. Ruland, Matthew A. McCoyd, José Biller

**Affiliations:** ^1^Department of Neurology, Stritch School of Medicine, Loyola University Chicago, Maywood, IL, USA

**Keywords:** direct funduscopy, ophthalmoscope, basic competency, milestones, clinical skills

## Abstract

**Objective:**

To determine the performance of direct funduscopy (DF) as part of the initial clinical assessment among different faculty physicians and residents from internal medicine, emergency medicine, and neurology (N).

**Methods:**

Retrospective study of 163 randomly reviewed charts of patients (>18 years) presenting either to the ED, inpatient units, or outpatient clinics from January 2001 to July 2013, with corresponding ICD-9 codes for headaches, altered mental status, and visual changes.

**Results:**

Although the Neurology Service was the one who performed most DF upon initial evaluation, DF is infrequently done throughout services independent of inpatient or outpatient location. Two thirds of the patients (66%) presenting with visual symptoms had evaluation done by Ophthalmology, which in some instances contributed to the final diagnosis.

**Conclusion:**

A more robust teaching of DF should be included among the basic clinical competencies during Medical School and Neurology Residency training.

## Introduction

The Association of University Professors of Ophthalmology (AUPO) has identified direct funduscopy (DF) as a basic competency, which should be part of all physicians’ repertoire. The Neurology Milestones Project used in evaluation of resident physicians in the context of their participation in Accreditation Council for Graduate Medical Education (ACGME) – accredited programs (1) listed, “visualizes papilledema” as a level 3 competence in the Neurological Examination – Patient Care 2 category.

Originally developed in 1851 by Hermann von Helmholtz (2), DF is performed using a direct ophthalmoscope to look at the fundus of the eye to examine posterior structures, including the optic disk, retinal arteries, retinal veins, retina, and macula. Although it has undergone many changes since its advent, the purpose remains the same. The posterior chamber may demonstrate signs of disease processes locally, such as a retinal hemorrhage, systemically, such as hypertension or cholesterol emboli, or in the nervous system, such as optic neuropathy or idiopathic intracranial hypertension. There are published reports suggesting that funduscopy is infrequently performed (3). In one study, one-third of patients screened with ocular examination, including funduscopic examination, were found to have subclinical ocular disease as part of a chronic disease process (4). With this in mind, it is disturbing that the funduscopic examination has been shown to be one of the clinical skills physicians are least confident performing (5). In not performing DF, valuable clinical information may be missed.

### Objective

To assess the frequency of DF as part of the initial clinical assessment by faculty physicians and residents from internal medicine (IM), emergency medicine (EM), and neurology.

## Materials and Methods

We retrospectively reviewed charts of 200 consecutive and randomly selected adult patients >18 years or older presenting to our emergency department (ED), inpatient units, or outpatient clinics from January 2001 to July 2013. Patients were identified using International Classification of Diseases 9th Revision (ICD-9) codes corresponding for headaches (784.0), visual symptoms-diplopia (368.2), blurry vision (368.8), visual loss (369.9), and altered mental status (AMS) (780.97). Thirty-seven charts were miscoded and were removed from our data pool, giving a total of 163 patients. Abstracted data included patient demographics, history of essential hypertension and diabetes mellitus, specialty of the physician performing the initial physical examination, whether or not DF was documented during the initial assessment, DF results from the ophthalmology service when present and final diagnoses.

## Results

Table 1 shows baseline patient characteristics. The mean age was 53 years (range 16–91 years) and 53% were women. Seventy-nine patients were initially seen as inpatients, 59 in the outpatient clinic, and 25 while in the ED. Visual complaints were the presenting problem in 50% of cases, headaches in 24.5%, and AMS in 25.2%. History of hypertension was present in 45% of patients, diabetes mellitus in 23%, and both hypertension and diabetes in 15%. Twenty-five percent of patients with headaches, 5% with AMS, and 26% with primary visual symptoms had DF upon initial evaluation. Table 2 shows performance of DF according to service.

**Table 1:** **Patients characteristics at initial encounter**.

	Total (*n* = 163)
**Age, mean (range)**	53 (16–91)
**Sex**	
Men (%)	77 (47)
Women (%)	86 (53)
**Patients per service**	
Internal medicine (%)	98 (60)
Emergency medicine (%)	25 (15)
Neurology (%)	40 (25)
**Setting**	
Hospital (%)	79 (48)
Clinics (%)	59 (36)
Emergency department (%)	25 (16)
**Presenting problems**	
Altered mental status (%)	41 (25)
Headaches (%)	40 (25)
Visual complaints (%)	82 (50)
**Comorbidity**	
HTN (%)	74 (45)
DM (%)	41 (23)
HTN + DM (%)	37 (15)

*HTN, hypertension; DM, diabetes mellitus*.

**Table 2:** **Frequency of DF according to Service**.

	Total patients per service	Number of direct funduscopy	% of patients with direct funduscopy
Internal medicine	98	11	0.11
Inpatient	47	6	
Outpatient	51	5	
Neurology	40	17	0.43
Inpatient	26	12	
Outpatient	14	5	
Emergency medicine	25	5	0.2

Ophthalmology was consulted in 53.4% of cases where visual symptoms were the primary symptom, 12.5% of headache patients, and none of the AMS cases. In the 21 patients presenting with visual symptoms where DF was performed by the primary service, 66.6% of those patients were also seen by Ophthalmology. Of the 10 headache patients where DF was performed, 40% had subsequent evaluations by Ophthalmology.

## Discussion

Our retrospective chart review shows that DF was infrequently performed. Some residency Program Directors believe that less than half of their residents meet competence with DF upon completion of Medical School (6, 7). Reports on the frequency of DF in practice vary according to the specialty, training level achieved, and encounter type. Dalay et al. (3) audited the charts of 92 cases and reported that only 18% of patients had DF as part of their acute medical assessment. Post graduate physicians (years 1–5) were surveyed and their perceived competency performing DF was directly proportional to their years in practice. The majority felt that more training was required (7). A similar survey by Roberts et al. found that only 3 of 41 postgraduate year 1–9 physicians working in Geriatrics and General Medicine routinely perform DF. A survey showed that only 18 (44%) of general and geriatric physicians were confident performing DF; 34 (83%) felt they would benefit from more training; 30 (73%) felt that they had insufficient training on DF; and most (97%) believed their DF skills could be improved. Reasons given for not performing DF include insufficient time, inadequate skills, lack of available equipment, and belief that DF is not useful. The authors concluded that training issues and encouragement to perform DF needs to be addressed before DF becomes a forgotten art (8). The Tendon Hammer, Ophthalmoscope, and Stethoscope (TOS) study was implemented following a serious unexpected incident occurred after missed papilledema at a large teaching hospital in the United Kingdom. The neurological examination by medical staff was evaluated using a patient assessment scoring system. Of 45 patients discharged from a neurology ward at one hospital over a 4-month period after a median hospital stay of 2 days, 100% recalled being examined with a tendon hammer, 97.8% with an ophthalmoscope, and 86.7% with a stethoscope (9).

In our study population, there is insufficient documentation to determine why initial DF was not performed. It is plausible that the reasons are similar to those reported by Roberts et al. (8). Documentation may have also affected the rates of DF found during our chart review.

A remedy could be increased training and exposure to ophthalmoscopy. In a 2006 study of medical students, greater training and exposure prompted greater use of and comfort with DF (10). Additional studies are needed to identify barriers to and improve DF performance.

Different factors may have played a role in the disproportionate percentages of DF performance among different subspecialties. Wall-mounted ophthalmoscopes are readily available in most offices and ED rooms in our center. However, not all inpatient wards are equipped with such tools. Therefore, the outpatient setting may be more conducive to DF due to greater ability to control ambient lighting and access to equipment. However, examination location did not influence performance of DF in our study. From the EMR, it is difficult to assess why DF was not performed as that portion of clinical reasoning was not documented in the chart.

## Conclusion

Our study shows that DF is infrequently performed during the initial physical examination by EM, IM, and neurology physicians in our Medical Center. Although we cannot speculate as to the reasons why, our data stress the importance of including DF among the basic clinical competencies during Medical School and Neurology Residency training. Moreover, we suspect our findings are probably widespread. Future studies should continue to focus on these three disciplines due to their likelihood of encountering patients presently with a variety of disease processes that could affect the fundus.

## Conflict of Interest Statement

The authors declare that the research was conducted in the absence of any commercial or financial relationships that could be construed as a potential conflict of interest.
